# On the Automaticity of Familiarity in Short-term Recognition: A Test of the Dual-Process Assumption with the PRP Paradigm

**DOI:** 10.5334/joc.21

**Published:** 2018-03-23

**Authors:** Klaus Oberauer

**Affiliations:** 1 University of Zurich, CH

**Keywords:** Recognition, working memory, psychological refractory period, dual-task, dual-process, familiarity, recollection

## Abstract

Dual-process models of recognition often assume that one retrieval process, generating a familiarity signal, is automatic, whereas the other, recollection, is controlled. Four experiments are presented to test for automaticity of familiarity in a short-term recognition task. The experiments use the Psychological Refractory Period (PRP) paradigm to assess whether familiarity requires central processing capacity. Task 1 was an oral tone-classification task. Task 2 was a local-recognition task, in which participants decided whether a probe matched a particular item in the memory set, identified by its screen location. Intrusion probes, matching an item of the memory set in a different location, were slower and more difficult to reject than new probes. The size of this intrusion cost reflects the influence of familiarity on recognition. In all four experiments the size of the intrusion cost was additive with the stimulus-onset-asynchrony (SOA) of Task 1 and Task 2, demonstrating that extraction of familiarity requires central capacity. In addition, Experiment 2 showed additive effects of memory set size and serial position with SOA, confirming that recollection, too, requires central capacity. Experiments 3A and 3B compared a condition including new probes to one including only positive and intrusion probes; in the latter condition the familiarity signal was completely uninformative. Participants showed some ability to reduce the influence of familiarity when it was completely uninformative, but only when they were explicitly told to do so (Experiment 3B). To conclude, by one criterion familiarity is a controlled process: It demands central processing capacity. It might also be controlled by another criterion: People can intentionally reduce the influence of familiarity on recognition decision, but they fail to do so spontaneously even when it would be advantageous. All raw data are available on the Open Science Framework: osf.io/7pr72.

Dual-process models of recognition assume that recognition decisions draw on information from two processes, referred to as *familiarity* and *recollection* (Diana, Reder, Arndt, & Park, 2006; Jacoby, 1991; Mandler, 1980; Oberauer, 2008; Yonelinas, 2002). Familiarity refers to memory for the recent occurrence of individual stimuli, without information about their episodic context. Its subjective experience is often described as knowing that one has recently encountered a stimulus, without remembering the episode. For instance, a person in a recognition experiment might know that a probe word had occurred on the study list without remembering any contextual details, such as which other words they have studied before or after, or which thoughts they had when studying the word – the probe word simply feels familiar. In contrast, recollection refers to memory of the recent occurrence of a stimulus in a particular context. Subjectively it is described as remembering the episode in which the stimulus has previously occurred, including some of its context. For instance, a person might remember that they have read the probe word in the upper left corner of the computer screen, and that they have thought about whether it could be linked to the preceding study word.

Dual-process theories of recognition vary in the degree to which they assume qualitative differences between familiarity and recollection as retrieval processes. A strong version of the dual-process idea is that familiarity and recollection refer to qualitatively different processes of retrieval that result in different cognitive states and feed into different decision processes. For instance, Yonelinas (1994) describes familiarity as a continuously varying memory signal feeding into a signal-detection process for discriminating old from new probes, whereas recollection is an all-or-none process that, if successful, results in a high-confidence judgment that the probe is “old”. A weaker version describes the difference between familiarity and recollection as one of the information that is retrieved. For instance, Wixted and Mickes (2010) proposed a dual-process model in which familiarity and recollection form two dimensions of continuously varying memory strengths, which can be combined into a single signal reflecting the evidence from memory that the probe is old. Similar models have been proposed by Rotello, Macmillan, and Reeder (2004) for recognition from episodic memory, and by Oberauer (2008) for recognition from working memory. Although often referred to as dual-process theories, these theories could be interpreted as assuming a single retrieval process that extracts evidence from two kinds of information in memory: The strength of the probe’s representation in memory – yielding the familiarity signal – and the links of the probe’s representation to elements of the relevant study context – yielding the recollection signal.

If familiarity and recollection actually reflect different processes, then these processes must differ in some way that goes beyond the information they retrieve. One potential difference that has been assumed in some dual-process theories is that the accrual of familiarity is an automatic process, whereas recollection is a controlled process (Jacoby, 1991).

## Does Familiarity Arise Through an Automatic Retrieval Process?

Automaticity is characterized by several attributes that do not always occur together (for a review see Neumann, 1984). Two key attributes are: (1) Automatic processes proceed without being intended, and as such, can operate against an intended mental process or action – for instance, word reading in the Stroop task (MacLeod, 1991). (2) Automatic processes are usually assumed to operate without capacity limits, such that they can run in parallel with other processes without suffering performance costs.

There is evidence that familiarity is automatic in the first sense: Familiarity of a probe influences recognition decisions even when that influence is detrimental to performance. For instance, it is harder to reject a negative probe (i.e., a probe not matching any item in the memory set relevant for the recognition decision) when a stimulus matching the probe has been a member of the relevant memory set in the previous trial (Atkinson, Herrmann, & Wescourt, 1974; Monsell, 1978; Nelson, Reuter-Lorenz, Sylvester, Jonides, & Smith, 2003), or when the probe matches a member of a memory set declared as irrelevant (Göthe & Oberauer, 2008; Oberauer, 2001). Whereas these findings imply that the influence of familiarity cannot be completely avoided in a recognition task, they do not show that people entirely lack control over its influence. Experiments 3A and 3B in the present series will make a first attempt to investigate this issue.

My main goal is to answer the question whether familiarity is automatic in the second sense: Does the extraction of a familiarity signal from memory rely on a limited processing capacity? Little is known about this aspect of the automaticity of familiarity.

To assess whether a process requires a limited processing capacity, we can measure whether it competes with other processes known to rely on that capacity. However, not every instance of dual-task costs reflects competition for a limited capacity. Dual-task costs can arise from various sources that are not reasonably described as capacity-limiting, such as conflict between incompatible demands on sensory systems or motor effectors (Neumann, 1984; Pashler, 1994), or strategic postponement of a task to comply with instructions (Meyer & Kieras, 1997). When these sources of dual-task costs are carefully ruled out, there remains evidence for a limit on the simultaneous execution of any two so-called *central* processes, which effectively acts as a strong, though not entirely immutable, bottleneck (Pashler, 1994). As such, it can reasonably be described as a processing capacity limit. Hence, we can specify the question at hand more precisely: Does the generation of a familiarity signal in recognition require central processing capacity?

There are competing theoretical characterizations of this capacity: For a long time, the dominant view has been that it is a structural bottleneck that can only carry out one central process at a time (Pashler, 1994; Van Selst, Ruthruff, & Jonston, 1999). Another view is of a limited processing resource that can be shared between two parallel processes, but at the price of slowing down each of them (Navon & Miller, 2002; Tombu & Jolicoeur, 2003). A third proposition is that the limit on central processes reflects a difficult-to-overcome setting of control parameters that serializes central processes to protect the system from cross-talk (Göthe, Oberauer, & Kliegl, 2016; Logan & Gordon, 2001). Here I will use the term *central capacity* simply to refer to the mechanism that constrains the parallel execution of central processes, remaining neutral about its nature.

There is also no agreement yet on what counts as a central process. Response selection – the choice of a response that is appropriate to a given stimulus or situation according to the current task set – has been identified unambiguously as a central process. Most pertinent to the present question, there is convincing evidence that retrieval from long-term memory is a central process: It is very difficult, and perhaps impossible, to use two retrieval cues in parallel (Rickard & Bajic, 2004), or to retrieve two responses to the same retrieval cue in parallel (Nino & Rickard, 2003; Strobach, Schubert, Pashler, & Rickard, 2014). Moreover, a concurrent response-selection demand from a second task has been found to delay retrieval (Carrier & Pashler, 1995). The study of Carrier and Pashler (1995) used the Psychological Refractory Period (PRP) paradigm, which I will rely on in the present experiments; therefore I next review their study in some detail.

## The Psychological Refractory Period Paradigm Applied to Memory Retrieval

**Figure 1:**
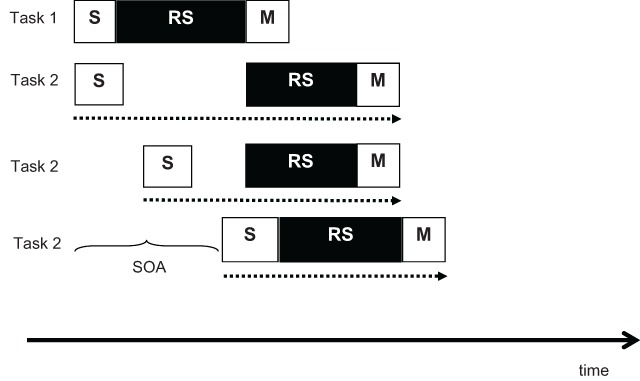
Illustration of the standard explanation of the PRP effect. The top two rows show the scheduling and durations of the processing stages (S = sensory, RS = response selection, M = motor execution) for Task 1 and Task 2, respectively, at SOA = 0: The central response-selection stage of Task 2 is delayed until the central process of Task 1 is completed, enforcing a delay (slack time) that is added to the RT of Task 2. The bottom two rows show task-2 processes at increasingly longer SOAs: As the SOA increases, the delay decreases, resulting in shorter task-2 RTs.

The PRP paradigm is a tightly controlled dual-task paradigm in which participants carry out two tasks, each requiring a speeded response to an imperative stimulus, and the stimulus-onset asynchrony (SOA) between the stimuli for Task 1 and Task 2 is varied. As the SOA is shortened, the temporal overlap between the two tasks increases, so that any competition for a shared processing capacity is expected to lead to increasing delays in one of the tasks (see Figure 1). Because participants are instructed to give Task 1 priority and always respond to it first, the delay is usually confined to Task 2. The typically observed effect is that response times (RTs) to Task 2 are increasingly delayed as the SOA is shortened. This *PRP effect* demonstrates that at least one process in Task 2 competes with a process in Task 1. The competition is resolved in favor of Task 1, so that the competing process of Task 2 is delayed.

**Figure 2:**
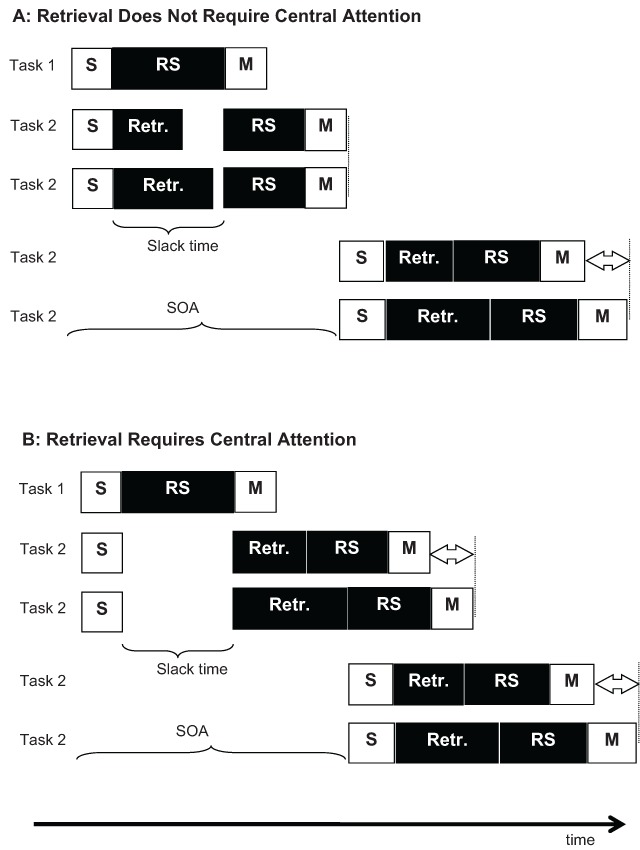
Hypothetical scheduling of processes for a retrieval task as Task 2 in the PRP paradigm. **A:** When retrieval does not require the bottleneck, it can be completed in the slack time at short SOAs, but not at long SOAs. Therefore, differences in retrieval duration between two conditions translate into RT differences at long SOAs (bottom two rows) but not short SOAs (rows 2 and 3). **B:** When retrieval requires the bottleneck, it can commence only after the slack period. Therefore, differences in retrieval duration manifest themselves fully in RT differences regardless of SOA.

Carrier and Pashler (1995) had participants do a tone-classification task as Task 1, and a memory-retrieval task as Task 2. Finding a PRP effect for Task 2 does not demonstrate that memory retrieval requires central capacity, because the memory task inevitably involves giving a response, so the retrieval process is followed by a response-selection stage, which is expected to be delayed when it competes with response selection for Task 1. To assess whether memory retrieval itself is delayed, the authors relied on the *locus-of-slack* logic, illustrated in Figure 2. The processes of Task 2 are subdivided into four successive stages, sensory (identification of the retrieval cue), memory retrieval, response selection, and motor execution. Any task-2 processing stage requiring central capacity is postponed until the central (response-selection) process of Task 1 is completed, whereas all preceding non-central stages can proceed in parallel with Task 1 without delay. Therefore, as the SOA is shortened, response selection of Task 2 is increasingly delayed, leaving an increasingly large temporal slack between perception (a non-central stage) and response selection. If memory retrieval does not require central capacity, it can use this slack time (Figure 2A). In contrast, if memory retrieval also requires central capacity, it is delayed until the end of task-1 response selection, and the slack interval remains unused (Figure 2B).

The two scenarios lead to different predictions about how a manipulation of the duration of memory retrieval interacts with SOA. If memory retrieval does not rely on central capacity, the effect of that manipulation is absorbed into the slack interval at short SOAs: Regardless of whether retrieval is fast or slow, task-2 response selection commences once task-1 response selection is finished, and therefore, the duration of retrieval has no effect on the RT of Task 2. Therefore, the manipulation of retrieval duration should have a smaller (and in the limit, vanishing) effect at shorter SOAs. In contrast, if memory retrieval relies on central capacity, any increase of its duration results in a concomitant delay of all subsequent processes, and therefore fully translates into an increase of RT, regardless of SOA. Therefore, the effect of a manipulation of retrieval duration is predicted to be additive with SOA. Across two experiments, using two memory tasks (probed recall and item recognition) as memory tasks, Carrier and Pashler found additive effects of SOA with the number of study repetitions of the retrieved material, a variable assumed to affect retrieval duration. This result supports the scenario depicted in Figure 2B, in which memory retrieval relies on central capacity.

A subsequent study by Green, Johnston, and Ruthruff (2011) questioned this conclusion. They carried out a PRP experiment very similar to that of Carrier and Pashler, with item recognition as Task 2, but changed a few features of the experiment to facilitate parallel processing. They observed the interaction of SOA and the number of study repetitions predicted on the assumption that memory retrieval does not require central attention. Green and colleagues discuss several reasons why their results might differ from those of Carrier and Pashler. One of them is that, because Green et al., put more emphasize on the speed of responding to the memory task, their participants relied more on familiarity, and less on recollection, than those of Carrier and Pashler. This possibility, together with the assumption that extraction of a familiarity signal does not require central capacity, whereas recollection does, could explain the discrepant findings. This speculation was one motivation for the present investigation.

## The Present Experiments

I carried out four experiments testing the hypothesis that familiarity arises from an automatic retrieval process that does not require central capacity, whereas recollection relies on central capacity. Experiments 3A and 3B also served to test the hypothesis that people have control over how strongly familiarity influenced their recognition decisions.

**Figure 3:**
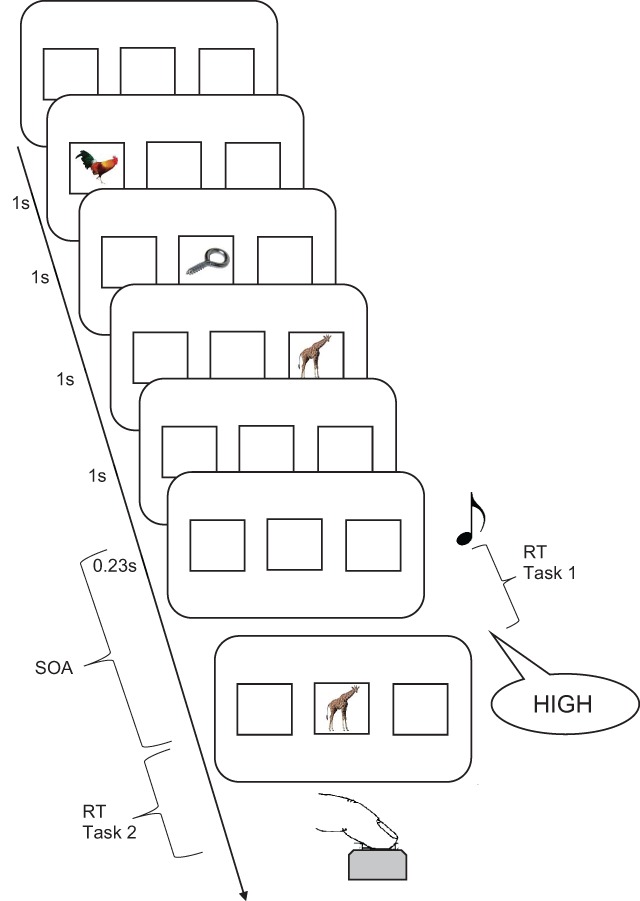
Flow of events in a trial of Experiment 1. The stimulus-onset-asynchrony (SOA) was varied (0.1, 0.3, 0.6, or 1.2s). The memory probe shown is an intrusion probe.

The experiments used the PRP paradigm, with a tone discrimination task as Task 1, and a local-recognition task as Task 2 (see Figure 3). The local-recognition paradigm (Oberauer, 2003) is a task for investigating recognition in short-term or working memory. Participants encode a short list of stimuli, each of which is presented in a different location on the screen. At test, a probe is presented in one of the locations, and participants decide whether it matches the list item they remember for that location. Three kinds of probes can be distinguished: Positive probes match the item in the probed location; new probes don’t match any list item; intrusion probes match a list item in a different location. The local-recognition task is suited for separating familiarity and recollection, because the intrusion probes set familiarity and recollection into conflict: An intrusion probe matches an item in the currently relevant memory set, and hence generates a familiarity signal as strong as that for a positive probe. Only by recollection of the relation between items and their context – in particular, their spatial location – can an intrusion probe be rejected. The ability to discriminate between positive and intrusion probes can therefore be used as a relatively pure index of recollection. A previous study with the local-recognition task, analyzing serial-position effects for the three probe types, has shown that a dual-process model explains the data from this task better than a single-process model (Oberauer, 2008).

An assumption that is important for the predictions derived below is that the two retrieval processes – accrual of familiarity and recollection – proceed at least partially in parallel. This assumption is shared by most dual-process theories of recognition (for a review see Yonelinas, 2002). This assumption is supported by studies using the signal-to-response method to track the time course of familiarity and recollection in recognition tasks (for studies using short-term recognition paradigms similar to the one used here, see Göthe & Oberauer, 2008; McElree & Dosher, 1989; Öztekin & McElree, 2010). In these studies, participants were given response signals at variable intervals after probe presentation, upon which they had to immediately make a recognition decision. At the shortest response intervals, accuracy is close to chance, and it increases with longer intervals as more information from memory becomes available. The important observation for the present discussion comes from comparing the time course of accuracy of new probes and of intrusion probes. Compared to new probes, intrusion probes produce a particularly high false-alarm rate at short intervals; at longer intervals, the false-alarm rate of the two probe types converges to a low level. This pattern can be explained by assuming that familiarity starts to accumulate earlier, generating a tendency to accept intrusion probes at short intervals. At a later point in time, recollection sets in and yields strong evidence for rejecting intrusion probes, which increasingly overcomes the misleading familiarity signal at longer intervals. Göthe and Oberauer (2008) fit the data from their experiment with process models incorporating these assumptions.

**Figure 4:**
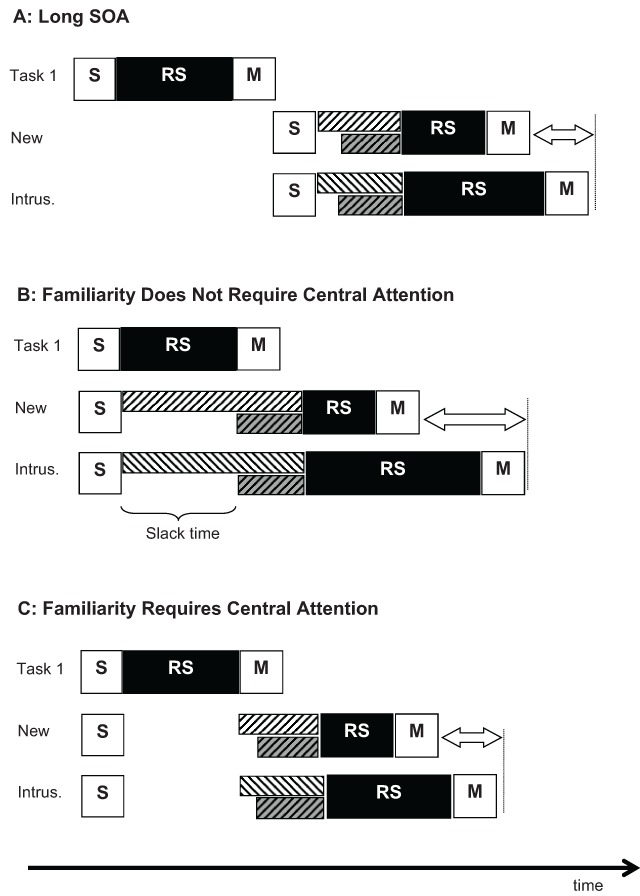
Two hypotheses about familiarity and recollection in the PRP paradigm. **A:** With a long SOA, no task-2 process is delayed by competition for the bottleneck. After sensory processing of the probe, familiarity (white striped bars) starts immediately. Recollection (grey striped bars), assumed to be a slower process, follows with a short delay. Response selection takes longer for intrusion probes than new probes due to the need to resolve the conflict between familiarity and recollection. **B:** Short SOA, scenario in which familiarity does not require the bottleneck. Familiarity receives a head start, accumulating more information before recollection sets in. This facilitates response selection for new probes but exaggerates the conflict for intrusion probes, resulting in a larger intrusion costs on task-2 RTs. **C:** Short SOA, scenario in which familiarity requires the bottleneck. Both familiarity and recollection are delayed equally, so that the intrusion cost is independent of SOA.

The logic of the predictions for the present experiments is illustrated in Figure 4. At a long SOA, all processes of the memory task (Task 2) proceed without delay (Figure 4A). Accrual of familiarity starts somewhat earlier than recollection, and after recollection commences, the two retrieval processes run in parallel. In the case of a new probe, familiarity and recollection provide converging evidence for rejecting the probe, making the decision easy; therefore, the response-selection stage is relatively short. In the case of an intrusion probe, familiarity provides evidence for accepting the probe, whereas recollection provides evidence for rejecting it. Resolving this conflict takes more time, resulting in a longer response-selection stage. As a consequence, correct rejection of intrusion probes takes longer than that of new probes.

Figure 4B illustrates the case of a very short SOA on the assumption that familiarity does not require central capacity, whereas recollection does. This means that the familiarity process already starts during the slack time. The shorter the SOA, the larger the head start of familiarity before recollection. This head start for familiarity is beneficial in the case of new probe, because it provides evidence for the correct decision, therefore speeding up response selection in comparison to the long SOA condition. However, in case of an intrusion probe the extended accumulation of familiarity exacerbates the misleading evidence that has to be overcome (with the help of recollection) to arrive at a correct decision, thereby prolonging response selection. As a consequence, the intrusion cost – the difference in RTs between intrusion probes and new probes – is predicted to increase at shorter SOAs. To the extent that the prolonged accumulation of familiarity during the slack period increases the chances that the familiarity signal exceeds the evidence from recollection, a larger intrusion cost in terms of errors (false alarms) is also to be expected. In sum, the assumption that recollection requires central capacity, but familiarity does not, entails the prediction of an over-additive interaction between SOA and the type of negative probe (new vs. intrusion).

Figure 4C shows the case of a very short SOA on the assumption that both familiarity and recollection must wait until the task-1 central process has finished. In this scenario, familiarity and recollection are postponed to the same extent, so that the SOA does not affect the balance between them. Therefore, RTs for both new and intrusion probes are prolonged by the same amount as the SOA is shortened, implying additive effects of SOA and the type of negative probe.

As mentioned above, these predictions hold if accrual of familiarity and recollection proceed in parallel, so that a head start for familiarity implies that familiarity accumulates for a longer time, and therefore has a stronger influence on the decision. The predictions also hold for a model in which the accrual of familiarity proceeds as long as it is not interrupted by the onset of recollection, or by the completion of recollection. The predictions do not hold, however, in a model such as the one proposed by Atkinson and Juola (1973), in which first only evidence from familiarity is considered, and only if that is inconclusive – familiarity falling between an upper threshold for acceptance and a lower threshold for rejection – a recollection process is initiated. In that model, a head start for familiarity would speed up recognition overall, but not shift the weight of evidence between familiarity and recollection. I will return to this possibility in the context of Experiments 3A and 3B. For the first two experiments, I will focus on the predominant class of dual-process models in which the two forms of retrieval run in parallel.

For completeness, we should consider the possibility that both familiarity and recollection do not require central capacity. In that case, at a short SOA both retrieval processes could run in parallel with the central stage of Task 1, using the slack time, effectively postponing the onset of response selection relative to that of both familiarity and recollection. This scenario would predict that any manipulation affecting the duration of recollection (or of both retrieval processes) – for instance, a manipulation of memory set size – should be absorbed into the slack at a short SOA. I will test this prediction in Experiment 2.

## Experiment 1

Experiment 1 used a tone-discrimination task with a vocal response as Task 1, and a local-recognition task with a manual response as Task 2. Memory items were three pictures of concrete objects. The combination of an auditory-vocal Task 1 with a visual-manual Task 2 has been shown to facilitate parallel processing (Göthe et al., 2016; Hazeltine, Ruthruff, & Remington, 2006), and the choice of visual memory materials reduces the chance of representational interference with the auditory and verbal representations involved in Task 1. Green et al. (2011) have argued that the evidence for a capacity limit for retrieval in the experiments of Carrier and Pashler (1995) could have been due to their choice of stimulus-response combinations disadvantageous for parallel processing. If that is the case, the present experiment should provide a good opportunity for obtaining evidence that familiarity proceeds in parallel with the central stages of Task 1.

This experiment was pre-registered at the Open Science Framework (Oberauer, 2016). Unless explicitly stated, all method decisions and analyses conform to the preregistration document.

### Method

**Participants.** Twenty-four students of the University of Zurich took part in a one-hour session in exchange for partial course credit or 15 Swiss Francs. Seven of them did not respond to Task 1, and therefore were excluded, leaving N = 17. For one further participant, data from the last 2 of 18 blocks were lost due to equipment failure.

**Materials.** For Task 1, participants heard either a high or a low tone (880 Hz vs. 220 Hz) for 226 ms. Stimuli for the memory task (Task 2) were 600 pictures of concrete objects. They were a subset of the 2400 pictures used by Brady, Konkle, Alvarez, and Oliva (2008) and downloaded from Tim Brady’s web page: https://bradylab.ucsd.edu/stimuli.html. For each memory set, three object pictures were chosen at random from the set of 600 pictures, with the constraint that no stimulus was repeated before the entire pool has been used once. The three pictures were displayed across a row of three frames on the screen. On 50% of all trials, the recognition probe was a positive probe, that is, a picture matching one of the memory items, and presented in the same frame in which that item had been presented in the memory set. On 25% of trials, the probe was a new object selected randomly from the pool, again with the constraint of not re-using any stimulus before the entire pool has been used once. On the remaining 25% of trials, the probe was an intrusion probe, that is, a picture from the memory set, presented in a different frame. Each serial position of the memory list was equally often chosen as the tested one. For intrusion probes, each of the two remaining (not-tested) list position was chosen equally often as its position of origin (i.e., the position in which the probe had been presented in the memory set).

**Procedure.** Each trial began with the presentation of the memory set: The three pictures were displayed sequentially across a row of three frames from left to right, for 1 s each, without pause between stimuli. One second after offset of the last picture, the tone for Task 1 was presented. Participants were instructed to say the German word for “high” or “low” as quickly as possible. Their responses were digitally recorded. Response onsets were timed off-line through an algorithm detecting a steep and sustained increase in the amplitude of the waveform.

The recognition probe for Task 2 followed the onset of the tone by a variable SOA: 0.1, 0.3, 0.6, or 1.2 s. The probe remained visible until participants responded, pressing the left arrow key for rejecting the probe (the correct response for new and intrusion probes) and the right arrow key for accepting it. Two seconds after the response, the memory display for the next trial was presented.

A completely balanced design required 96 trials, crossing 4 SOAs, 4 probes (2 positive, 1 new, 1 intrusion), 3 tested serial positions, and 2 serial positions of origin (for intrusion probes). This design was repeated 3 times, for a total of 288 trials. These trials were presented in random order, organized into 18 blocks of 16 trials each. Between blocks, participants were encouraged to take a brief break. Before the test trials there were 16 practice trials, selected at random from the 96 trial types of the basic design with the constraint that there were 4 trials for each SOA level.

### Results

The preregistration document specifies only the analysis of task-2 RTs, but the analysis of task-2 errors is equally important because the over-additive interaction predicted by the notion of capacity-free familiarity could emerge in either dependent variable, or both. Task-1 RTs are not relevant for the hypotheses in question but are briefly reported to provide a full picture of dual-task costs.

I pre-processed the RT data as follows: First, task-2 RTs of incorrect responses were removed. For Task 1, accuracy was not recorded because the task is trivially easy, and task-1 performance is of minor interest for the research question. Next, I removed as outliers any RT shorter than 0.2s, and any RT that exceeded a person’s mean RT in a SOA × probe-type design cell by more than 3 intra-individual standard deviations.

I analyzed the unaggregated task-2 RTs, as well as the proportion of correct responses in each design cell, with a Bayesian linear mixed model with SOA (4 levels) and probe type (new vs. intrusion) as independent variables. The BANOVA was run with the BayesFactor package (Morey & Rouder, 2015) in R (R_Core_Team, 2017). The BayesFactor package calculates the Bayes Factor (BF) for pairwise model comparisons. Each analysis started from the full model including fixed effects (2 main effects and one interaction) as well as random intercepts (i.e., individual differences in the grand mean) and random slopes for the two predictors (i.e., individual differences in the size of the main effects). In a first step I tested whether the inclusion of each random slope was warranted by comparing the full model against a model excluding the random slopes. Random slopes were maintained in the model if the BF in favor of the full model exceeded 1, and removed from the model otherwise; the resulting model became the full model for the next steps. In a second step I tested evidence for the interaction by comparing the full model to one with the interaction removed. The BF in favor of the full model reflects the evidence for the interaction; its reciprocal (i.e., the BF for the reduced model) reflects evidence against the interaction. In a third step, I tested each main effect by comparing an additive model including both main effects (but not the interaction) against a model in which the fixed main effect in question (but not its random slope) was removed. The BF in favor of the additive model is the evidence for the main effect. All models were run with the default priors on effect sizes proposed by Rouder, Morey, Speckman, and Province (2012) for ANOVA designs, using a scale factor of ½. With this scaling factor, most of the prior is concentrated on small and medium effect sizes.

**Figure 5:**
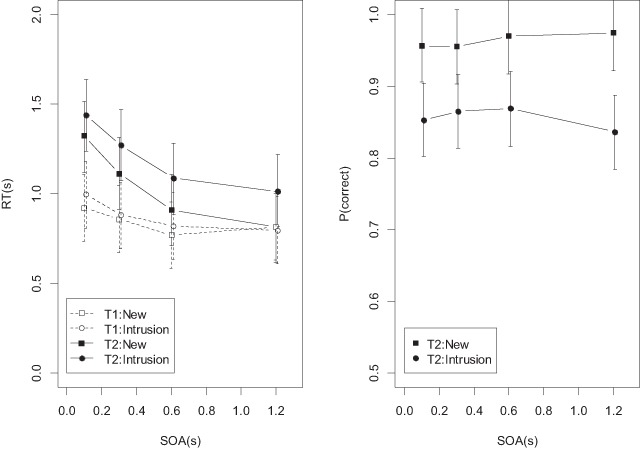
Mean RTs and accuracies from Experiment 1, new and intrusion probes. T1 = Task 1, T2 = Task 2. Error bars are 95% credible intervals of the posterior density of the mean, estimated from the full regression model.

**Task 2 (Recognition).** Mean RTs and proportion correct for the recognition task are plotted in Figure 5 (filled points). The RT data show a clear PRP effect, reflected in the main effect of SOA (BF = 8.7 × 10^13^). There was also strong evidence for an intrusion cost, reflected in the main effect of probe type (BF = 20.3). There was clear evidence against the interaction (BF = 0.0226 in favor, implying BF = 44.1 against the interaction).

For proportion correct, the evidence speaks against a main effect of SOA (BF = 0.14 in favor, implying BF = 6.8 against), but in favor of a main effect of probe type (BF = 9552). There was weak evidence against the interaction (BF = 0.5). Figure 5 shows a slight trend for the intrusion cost to become smaller at shorter SOAs, contrary to what is predicted from the assumption that recollection requires central capacity whereas familiarity does not.

**Task 1.** The RTs to Task 1 (unfilled points in the left panel of Figure 5) showed a shallow trend towards slower responses at short SOAs, which was statistically supported (BF = 230). There was no effect of probe type (BF = 0.19), and no interaction (BF = 0.06).

### Discussion

The results were unambiguously against the over-additive interaction of SOA and probe type that is predicted from the hypothesis that familiarity is independent of central capacity limits. There was also no hint of an under-additive interaction on RTs, as predicted if both familiarity and recollection were capacity free. The additive effects of SOA and probe type support the assumption that both familiarity and recollection rely on central processing capacity, so that they have to wait until the central processing stage of Task 1 has finished.

## Experiment 2

The second experiment repeats the design of the first, with two changes: For generality, I used words instead of pictures as memory items. In addition, I varied memory set size (2 vs. 4 words). Larger memory sets increase response times, and this effect is in large part due to the longer duration of retrieval (Shepherdson, Oberauer, & Souza, 2017). Therefore, we can use set size as a manipulation of the duration of the retrieval stage. One previous study testing visual working memory with an item-recognition test found an under-additive interaction of set size with SOA at a short but not a long retention interval (Magen, 2017), suggesting that under some circumstances retrieval might be capacity-free. In addition, with set size 4 it is possible to analyze serial-position effects. RTs and accuracies in local recognition are characterized by a roughly symmetrical U-shaped serial-position curve (Oberauer, 2003). A detailed analysis showed that the serial-position effect is an effect on the difficulty of recollection, not familiarity (Oberauer, 2008). Therefore, we can use the serial-position effect on RTs as a manipulation of the duration of recollection. According to the locus-of-slack logic (Figure 2), if retrieval does not require central capacity, the set-size effect should be at least partially absorbed in the slack, leading to an under-additive interaction of SOA and memory set size. Likewise, if recollection does not require central capacity, the serial-position effect should be absorbed in the slack, leading to an under-additive interaction of SOA with serial position. Experiment 2 provides an opportunity to test these predictions, in addition to the predictions pertaining to the SOA × probe-type interaction.

This experiment was not pre-registered; the analyses of effects of SOA and probe type still conform to the pre-registered plan for Experiment 1.

### Method

**Participants.** Twenty-four new students from the University of Zurich took part in a 1-hour session in exchange for partial course credit or 15 Swiss Francs. No participant was excluded from analysis.

**Materials and Procedure.** In most regards Experiment 2 was like Experiment 1, with the following differences: Instead of pictures, memory items were German words, sampled from a pool of 676 words (mostly nouns, plus a few adjectives, 4 or 5 letters long). Words were presented sequentially from left to right across a row of 2 or 4 boxes; regardless of set size, the row of boxes was centered on the screen.

The design for set size 2 crossed 4 SOAs with 4 probes (2 positive, 1 new, 1 intrusion), and 2 serial positions of test. The resulting 32 combinations were repeated twice, for 64 trials. The design for set size 4 crossed 4 SOAs with 4 probes, 4 serial positions of test, and 3 serial positions of origin of the intrusion probe. Each of the 192 combinations was presented once. The entire set of 256 trials was run in random order.

### Results

RT data were pre-processed as in Experiment 1. I analyzed the recognition data with three sets of Bayesian linear mixed models, one focusing on the interaction of SOA with probe type (new vs. intrusion), as in Experiment 1 (aggregating over set size), the second focusing on the SOA × set-size interaction (including all three probe types), and the third focusing on the SOA × serial-position interaction (including all three probe types, but only set size 4).

**Figure 6:**
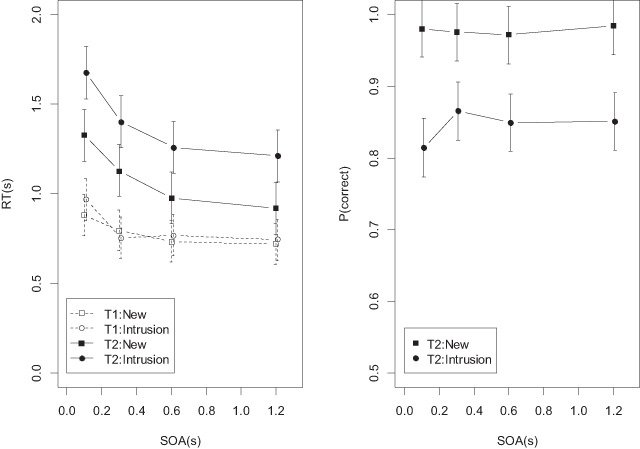
Mean RTs and accuracies from Experiment 2, new and intrusion probes.

**Task 2 (Recognition).** Figure 6 shows the mean RTs and proportion correct values for the analysis of SOA and probe type (new vs. intrusion). Recognition RTs once again showed a strong PRP effect (BF = 1.79 × 10^17^ for the main effect of SOA), and a substantial intrusion cost (BF = 1.68 × 10^5^ for the main effect of probe type). The evidence speaks strongly against the interaction (BF = 64 against). For accuracy, there was weak evidence against the main effect of SOA (BF = 0.3), and strong evidence in favor of a main effect of probe type (BF = 8172). There was weak evidence supporting the interaction (BF = 2.45). The right panel of Figure 6 shows that the interaction goes in the direction predicted by capacity-free familiarity: The intrusion cost was increased at the shortest SOA.

**Figure 7:**
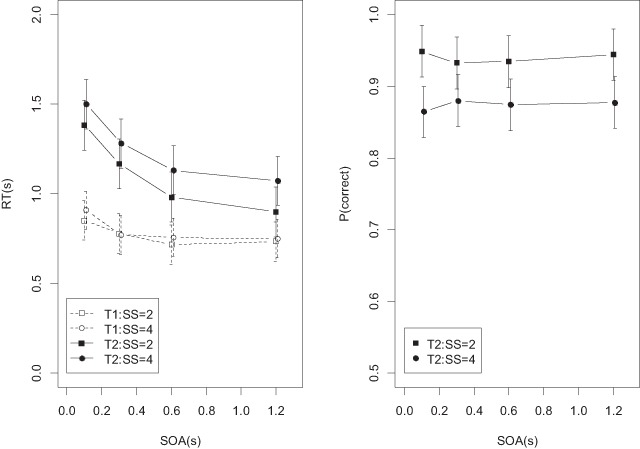
Mean RTs and accuracies of the recognition task in Experiment 2 as a function of set size, averaged over all probe types.

Figure 7 shows the data from the analysis of SOA and set size. In addition to the main effect of SOA on recognition RTs (BF = 7.32 × 10^202^) there was a main effect of set size (BF = 2.04 × 10^28^), and evidence against the interaction (BF = 124 against). For accuracy, there was evidence against the main effect of SOA (BF = 0.04), in favor of the set-size effect (BF = 2.16 × 10^13^), and modest evidence against the interaction (BF = 4.5 against).

**Figure 8:**
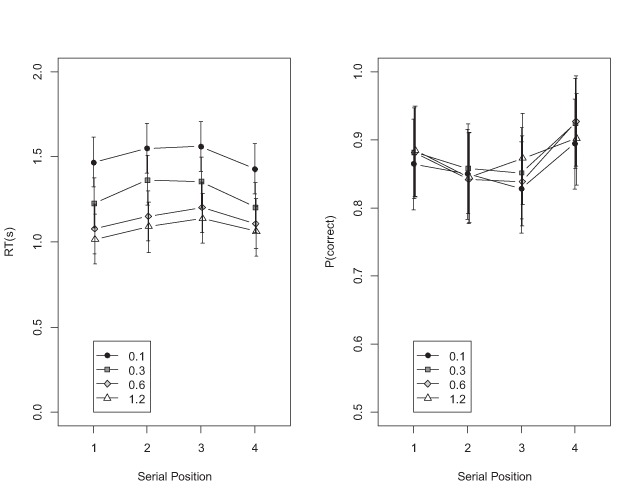
Mean RTs and accuracies of the recognition task in Experiment 2 as a function of serial position (set size 4), averaged over all probe types.

Figure 8 shows mean RTs and accuracies of the set-size 4 condition by SOA and serial position. For recognition RTs, besides evidence for the main effect of SOA (BF = 4.10 × 10^134^) there was also evidence for the effect of serial position (BF = 1.36 × 10^12^), reflecting the usual inverted U-shaped serial-position curves for RTs. Once again, the evidence was clearly against the interaction (BF = 2236 against). For accuracy, the evidence was against the main effect of SOA (BF = 0.01), in favor of the typical U-shaped effect of serial position (BF = 5482), and against the interaction (BF = 166,243 against).

**Task 1.** Analysis of task-1 RTs revealed no surprises. For all three analyses corresponding to the ones on task-2 data, there was evidence for a main effect of SOA, indicating a small dual-task cost on task-1 RTs (all BF > 10^21^). There was consistent evidence, though not always strong, against main effects of the other variables (probe type, set size, or serial position; all BF < 1), and against their interactions with SOA (all BF < 1).

### Discussion

In agreement with Experiment 1, the present experiment provided strong evidence against the interaction of SOA and probe type on recognition RTs. For accuracy, the results were more ambiguous, with a hint towards an over-additive interaction. Therefore, considering Experiment 2 on its own, we could not confidently rule out the hypothesis that the accrual of familiarity is capacity free. Taking both experiments together, the evidence is more strongly against this idea: Whereas in Experiment 2 the interaction of SOA and probe type on recognition accuracies trended in the predicted direction, in Experiment 1 it trended in the opposite direction. In both cases the Bayes Factors were in the range commonly regarded as weak evidence (Raftery, 1995). Most likely, these trends are just noise.

The additive effects of SOA and probe type are compatible with the notion that both familiarity and recollection require central capacity, but they could also be reconciled with the idea that they are both capacity free. Experiment 2 yielded additional evidence against the latter hypothesis. If both retrieval processes were capacity free, then any variable affecting retrieval duration – such as set size and serial position – should interact under-additively with SOA. This interaction was observed for neither set size nor serial position. These results decisively rule out the hypothesis that retrieval from working memory – including recollection – is a capacity-free process.

## Experiments 3A and 3B

I designed a third experiment to serve two goals. The first was to ask to what degree familiarity is automatic in the first sense discussed above: How much control do people have over the use of the familiarity signal in a recognition test? The second goal was to test whether a familiarity-based recognition process requires the bottleneck, on the assumption that familiarity precedes recollection (Atkinson et al., 1974). Participants worked on the local-recognition task in two conditions, administered in separate blocks. In the 3-probe condition they experienced a mixture of positive, new, and intrusion probes as in the preceding experiments. In the 2-probe condition they experienced only positive and intrusion probes. In the 3-probe condition, the familiarity signal is useful on a subset of the trials, because it helps rejecting new probes. In the 2-probe condition, familiarity is never useful, because it does not discriminate between positive and intrusion probes. If participants can control the influence of familiarity on recognition, they should rely on it less in the 2-probe condition than the 3-probe condition. Depending on whether familiarity and recollection run in parallel or sequentially, this should have different consequences.

In the parallel model considered so far, familiarity and recollection support each other for positive probes, and conflict with each other for intrusion probes. If familiarity is used more strongly in the 3-probe condition than the 2-probe condition, RTs should be faster for positive probes, and slower for intrusion probes, in the 3-probe than the 2-probe condition. In the serial model, in which familiarity precedes recollection, in the 2-probe condition participants could skip the processing stage evaluating familiarity, because it is completely uninformative. This should lead to faster RTs for both positive and intrusion probes in the 2-probe than the 3-probe condition.

If one of these two patterns of results is obtained, it is informative about the processing architecture (parallel vs. serial) of familiarity and recollection. In addition, if the evidence supports the serial architecture, we can use the PRP paradigm to test whether the familiarity-based stage requires the bottleneck. If it does not, the duration of this stage should be absorbed into the slack as the SOA is shortened. As a consequence, the RT difference between the 2-probe and the 3-probe condition should be diminished at short SOAs. In contrast, if the familiarity-based stage requires the bottleneck, the effect of probe mix (2-probe vs. 3-probe) should be additive with SOA.

### Method

**Participants.** Two samples (one each for Experiments 3A and 3B) of twenty-four students of the University of Zurich took part in a one-hour session in exchange for partial course credit or 15 Swiss Francs.

**Materials and Procedure.** Materials and procedure were identical to Experiment 1, except for the composition of probe types. The experiment was subdivided into four blocks, alternating between the 2-probe and the 3-probe condition; order of blocks was counterbalanced between participants. In the 3-probe condition, an equal proportion of trials had positive, new, or intrusion probes. I chose a larger proportion of new probes than in the previous experiments to increase the motivation to use familiarity in this condition. In the 2-probe condition, the new probes were replaced by intrusion probes, so that there were 1/3 positive and 2/3 intrusion probes. The high proportion of intrusion probes should strongly discourage the use of familiarity because it is misleading on intrusion probes. Participants were informed of the probe mix at the beginning of each block. Each block consisted of 72 test trials, preceded by 12 practice trials.

I ran this experiment in two versions differing in the instructions: Version A merely described the probe composition of the two kinds of blocks (2-probe vs. 3-probe blocks). Version B additionally explained that the feeling of familiarity can be used to reject new probes in the 3-probe blocks, but is not a useful cue for any recognition decisions in the 2-probe blocks. This explanation was intended to further motivate participants to try to control the use of familiarity for their decisions. Version A was pre-registered after completion of the first two experiments (Oberauer, 2017); Version B was carried out following the suggestion of a reviewer.

### Results and Discussion

**Figure 9:**
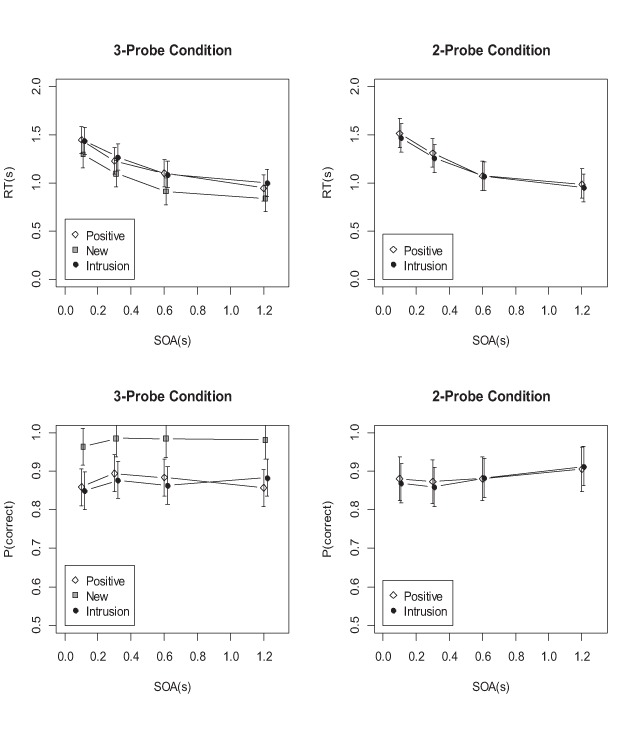
Mean RTs and accuracies of the recognition task in Experiment 3A.

**Figure 10:**
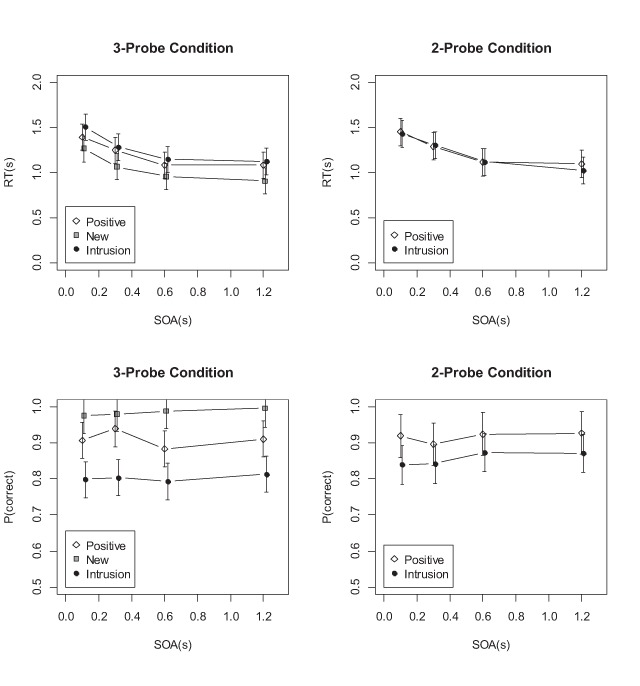
Mean RTs and accuracies of the recognition task in Experiment 3B.

RT data were pre-processed as in Experiment 1. The first set of analyses compared RTs and accuracies for positive and intrusion probes between the two probe-mix conditions. Figures 9 and 10 plot the mean RTs and accuracies for versions A and B, respectively.

The results of Version A are simple: The evidence was against any difference between the two conditions (BF against the main effect of condition = 21.6 for RTs, and 3.5 for accuracy; BF against the interaction of condition and probe type = 2.4 for RT, and 3.8 for accuracy). Probably participants did not control the degree to which they used familiarity for the recognition decision.

In contrast, the data from Version B – in which participants were explicitly instructed to not use familiarity in the 2-probe blocks – yielded some evidence for successful control of familiarity: There was strong evidence for the interaction of condition and probe for RTs (BF = 10.6), and weak evidence for the same interaction for accuracies (BF = 1.8). There was evidence against a main effect of condition on RTs (BF = 23 for the Null), but some evidence for a condition effect on accuracy (BF = 3.0). The pattern of these effects, just noticeable in Figure 10, is as predicted by the assumption that familiarity is used less in the 2-probe condition, assuming a parallel model: Compared to the 3-probe condition, in the 2-probe condition RTs to intrusion probes were faster, and RTs to positive probes were slower. In addition, in the 2-probe condition participants committed fewer false alarms to intrusion probes.

The results from the 3-probe conditions in both versions of Experiment 3 replicate Experiments 1 and 2: For recognition (Task-2) RTs, there was a main effect of probe type (new vs. intrusion, BF = 98.3/791.5 in Versions A/B), demonstrating that familiarity did influence recognition. This effect again did not interact with SOA (BF = 268.0/212.3 for the Null). For recognition accuracies, there was also a main effect of probe type (BF = 2318.8/88017.4) and no interaction with SOA (BF = 12.8/12.2 for the Null). RTs for the tone task (Task 1) showed no effects of probe type, SOA, or their interaction (all BF < .25). The additive effects of probe type and SOA on recognition RTs again corroborate the conclusion that familiarity requires the bottleneck, on the assumption that familiarity and recollection proceed at least partially in parallel.

## General Discussion

Is the accrual of familiarity in recognition an automatic process? The answer depends on what we mean by *automatic*. One criterion for an automatic process is that, in certain circumstances, it occurs without being intended. Prima facie evidence suggests that familiarity meets this criterion well, as it influences recognition decisions even when that is detrimental to performance, causing intrusion costs (Monsell, 1978; Oberauer, 2001). However, all previous experiments investigating intrusion costs have included trials with new probes, and these new probes could be rejected quickly and with high accuracy based on their lack of familiarity. Therefore, it could be argued that in these experiments relying on familiarity is to some extent useful. Perhaps participants can avoid using familiarity in a situation in which using familiarity is unambiguously detrimental. I created such a condition in the 2-probe blocks of Experiments 3A and 3B. When participants were merely told about the probe composition of each block (Version A), their performance in the 2-probe condition was indistinguishable from the 3-probe condition, implying that they did not reduce the use of familiarity even when it was entirely useless. However, in Version B, when the instruction explicitly told participants that they should not rely on familiarity in the 2-probe condition, they managed to reduce its influence – although the effect was small. To conclude, people can, to some extent, control the use of familiarity, although they do not do so spontaneously.

**Figure 11:**
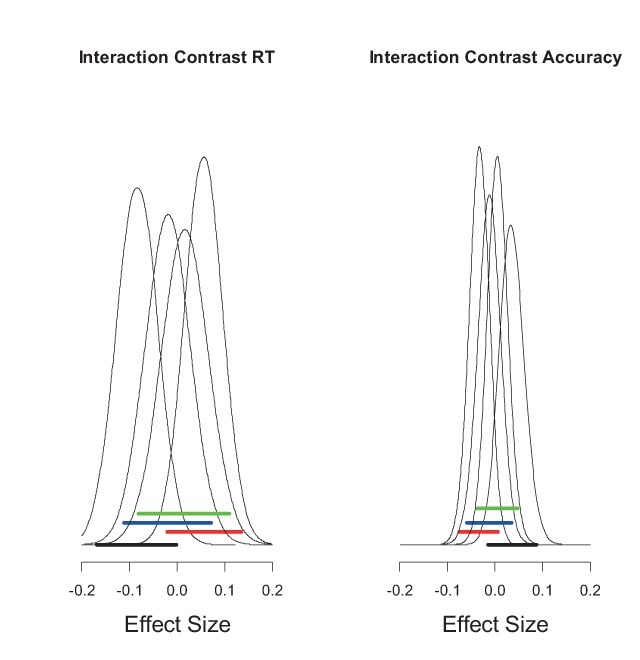
Posterior densities of the Probe Type × SOA interaction from the full linear model of the four experiments. Interaction contrasts were calculated as {(SOA = 0.1 & Intrusion) – (SOA = 0.1 & New)} – {SOA = 1.2 & Intrusion) – (SOA = 1.2 & New)}. The 95% credible intervals are given as thick horizontal bars (black, red, blue, and green for Experiments 1, 2, 3A, and 3B, respectively). The scale is the standardized effect size (i.e., Cohen’s d).

A second criterion is that an automatic process should not rely on any limited processing capacity. The main goal of the present work was to determine whether familiarity meets this second criterion. Research on dual-task costs has converged on the insight that there is a severe capacity limit on so-called *central* processes (Pashler, 1994). The second criterion can therefore be formulated more specifically as: Does familiarity depend on central processing capacity? The results of the four experiments reported here fairly unambiguously imply that it does: The intrusion cost was consistently additive with SOA, as predicted from the assumption that both familiarity and recollection require the bottleneck. Figure 11 provides a summary of this finding in the form of the posterior densities of the Probe Type × Interaction contrast. These posteriors are closely confined to a range of effect sizes around zero. These results confirm the conclusions from previous studies (Carrier & Pashler, 1995; Nino & Rickard, 2003; Rickard & Bajic, 2004) that retrieval in general – including familiarity and recollection – relies on central capacity.

This conclusion is at odds with the one by Green et al. (2011). Their finding of an under-additive interaction of SOA with memory strength suggests that at least one form of retrieval – perhaps familiarity – can proceed in parallel with the central stage of a concurrent task. In their critique of the experiments of Carrier and Pashler (1995) Green and colleagues argue that three features of their experiments could have caused the appearance of a capacity limit on retrieval: (1) Carrier and Pashler used tasks with stimulus-response modality mappings that make parallel processing difficult; (2) they required manual responses for both tasks, potentially creating motor interference; (3) they placed more emphasis on the speed of Task 1 than of Task 2. In the present experiments I avoided all three potential problems, and still found evidence that retrieval requires central capacity. In particular, the observation of additive effects of SOA with two manipulations of memory strength in Experiment 2 – set size and serial position – stand in contrast to the under-additive interaction of Green et al. (2011). One obvious difference between their experiments and the present ones is that they tested recognition from episodic long-term memory, whereas I tested recognition from working memory. It is conceivable that retrieval – or more specifically, extraction of a familiarity signal – from working memory, but not from episodic long-term memory, relies on central capacity. I see no theoretical reason to justify that assumption, but it might be worthwhile to test it anyway. The experimental design used here, combining the PRP paradigm with a recognition task in which familiarity can be brought into conflict with recollection, could be useful for that purpose.

If both familiarity and recollection underlie the central capacity limit, how can they run in parallel? The assumption of two parallel retrieval processes is difficult to reconcile with a structural bottleneck that permits only one process at a time. It is better compatible with a resource model of central processing capacity (Navon & Miller, 2002; Tombu & Jolicoeur, 2003), which allows parallel processes at the price of slowing them down. A third view of the limit on central processes is that it reflects control settings scheduling two processes in parallel to prevent cross-talk between two stimuli mapped onto two responses (Göthe et al., 2016; Logan & Gordon, 2001). In the case of familiarity and recollection, the risk of cross-talk is small because they belong to the same task, mapping the same stimulus to the same response – except, of course, for an intrusion probe, in which the stimulus is mapped to opposite responses. The intrusion cost can be interpreted as a manifestation of the cross-talk between the two retrieval processes. The control settings for recognition tasks might accept that price and schedule the two retrieval processes to run in parallel anyway. At the same time, to explain the PRP effect both the resource and the cross-talk interpretation would have to maintain that the central processes of the recognition task (Task 2) are scheduled to commence after the response-selection stage of Task 1 has finished. This assumption can be justified by the executive system’s tendency to avoid cross-talk between processes belonging to different tasks, or by the instruction to complete Task 1 before Task 2.

An alternative interpretation is that familiarity and recollection are not actually two different processes, and therefore do not compete for central resources at all. This consideration brings us back to the distinction in the introduction between two flavors of dual-process theories, one making a strong distinction between familiarity and recollection as qualitatively different process, and the other regarding them as merely two different sources of information retrieved from memory. In the latter view, there is a single retrieval process that delivers information about the recent encounter of a stimulus (i.e., familiarity) and about the relation of that stimulus to its context (i.e., recollection) in parallel. This view has the advantage of simplicity, as it does not have to assume that some central processes run in parallel whereas others have to wait for each other.

One motivation for the present endeavor was the search for a qualitative difference between familiarity and recollection, with one but not the other requiring a central processing resource. I failed to find evidence for that assumption. This does not mean that there is no qualitative difference in other regards, but so far none has been demonstrated. Therefore, the weaker interpretation of dual-process theories remains a viable alternative, and one could argue for it on the basis of parsimony.

Dual-process theories have been proposed in many other areas of psychology besides recognition, including reasoning, judgment and decision making, and social cognition (Evans, 2008). Often these theories include the assumption that one process is automatic, whereas the other is controlled. The analytical approach I applied here to short-term recognition could also be applied to test this assumption for dual-process theories in other areas.

## Data Accessibility Statement

All raw data are available on the Open Science Framework: osf.io/7pr72.
